# Involvement of Activin E depletion in metabolic dysfunction-associated steatohepatitis

**DOI:** 10.1016/j.bbrep.2025.102339

**Published:** 2025-11-04

**Authors:** Maho Sakaki, Tatsuya Shikata, Kensuke Aoki, Masaaki Takano, Akira Kurisaki, Masayuki Funaba, Osamu Hashimoto

**Affiliations:** a Laboratory of Veterinary Toxicology, College of Bioresource Sciences, Nihon University, Fujisawa, Kanagawa, 252-0880, Japan; b Faculty of Veterinary Medicine, Kitasato University, School of Veterinary Medicine, Towada, Aomori, 034-8628, Japan; c Graduate School of Biological Sciences, Nara Institute of Science and Technology, Ikoma, Nara, 630-0192, Japan; d Division of Applied Biosciences, Graduate School of Agriculture, Kyoto University, Kitashirakawa Oiwakecho, Kyoto, 606-8502, Japan; e Nagahama Institute of Bio-Science and Technology, Nagahama, Shiga, 526-0829, Japan

**Keywords:** Inhbe, MASLD, Insulin resistance, Adipose tissue, Obesity

## Abstract

Activin E is a liver-derived hepatokine belonging to the transforming growth factor-β superfamily, and it promotes energy expenditure by activating brown and beige adipocytes. Activin E also possesses anti-lipolytic activity. When Activin E knockout (KO) mice are fed a high-fat diet, energy storage in adipose tissue is impaired, leading to ectopic fat accumulation in the liver. In this study, we investigated the involvement of Activin E depletion in metabolic dysfunction-associated steatohepatitis (MASH), and we characterized the phenotype of Activin E-KO mice under a high-fat diet. Despite being protected from obesity, Activin E-KO mice developed pronounced hepatomegaly, hepatic triglyceride accumulation, and histological features consistent with MASH, including steatosis, ballooning degeneration, fibrosis, and increased hepatic crown-like structures. An RNA sequencing analysis showed a gene expression signature characteristic of MASH, including upregulation of inflammatory and fibrogenic pathways. In contrast, white adipose tissue mass and adipocyte size were markedly reduced, accompanied by elevated circulating non-esterified fatty acid and insulin concentrations, indicating adipose tissue dysfunction and insulin resistance. These features were observed even in the absence of obesity, indicating a lean-type MASH phenotype. Notably, Activin E-KO female mice showed delayed disease onset, suggesting estrogen-related protection. Our findings establish Activin E as a key regulator of adipose-liver metabolic communication and suggest that its loss promotes MASH via impaired lipid storage in white adipose tissue and an increased hepatic lipid burden. High-fat diet-fed Activin E-KO mice represent a novel lean-type MASH model and may serve as a useful platform for investigating hepatokine-targeted therapies.

## Highlights

• Activin E-KO mice on an HFD show liver enlargement and MASH-like features.
• Activin E-KO mice have less WAT fat and impaired insulin sensitivity.
• Female KO mice show milder liver damage than male KO mice.
• Activin E-KO mice are a lean-type MASH model.

## 1 Introduction

An increase in body fat, particularly visceral fat, due to environmental or genetic factors can induce insulin resistance and functional abnormalities in adipose tissue. This increase leads to elevated blood concentrations of free fatty acids and ectopic fat accumulation in tissues, such as muscle, and in organs, such as the pancreas. Steatotic liver disease (SLD), a condition characterized by neutral fat accumulation in the liver, has gained attention as a contributor to cardiovascular diseases, including cerebral and myocardial infarctions, and as a risk factor for cirrhosis and liver cancer. When SLD is accompanied by obesity, type 2 diabetes, or by two or more metabolic abnormalities (e.g., hypertension, visceral/abdominal obesity, elevated triglyceride concentrations, and low high-density lipoprotein cholesterol concentrations, it is diagnosed as metabolic dysfunction-associated steatotic liver disease (MASLD) [1]. Recently, the incidence of MASLD has increased compared with pre-pandemic levels, likely because of the increasing incidence of metabolic diseases, such as obesity and diabetes associated with sedentary behavior during the coronavirus disease 2019 pandemic [2,3].

SLD, which is common in patients with MASLD, may progress to metabolic dysfunction-associated steatohepatitis (MASH). MASH is a more severe condition involving inflammation and fibrosis that can lead to cirrhosis and liver cancer, where hepatocyte destruction results in loss of function.

MASLD and MASH are typically associated with obesity. However, recent studies have shown that these conditions can also occur in individuals with a non-obese phenotype, a condition often referred to as lean MASLD/MASH [4]. Notably, the prevalence of lean MASLD/MASH has been reported to be particularly high in Asian populations [5].

There is an urgent need for rapid diagnostic tools and effective treatments for MASLD/MASH, although no specific medication is currently available, and lifestyle modification remains the mainstay of treatment. However, the limited efficacy of current interventions indicates the importance of developing appropriate animal models of MASLD/MASH to support the discovery of novel therapies targeting adipose tissue dysfunction and metabolic dysregulation [6].

Activin E, which is specifically expressed in the liver, is a hepatokine belonging to the Activin superfamily [7,8]. Activin E increases energy metabolism by increasing UCP1 expression in brown adipocytes and beige adipocytes in white adipose tissue (WAT) in experiments involving transgenic mice that overexpress Activin E [9,10]. Furthermore, Activin E enhances UCP1 expression, promoting heat production and metabolic activity in brown adipocytes and fibroblast growth factor 21 [11], which improves insulin sensitivity and metabolism. These findings suggest the potential role of Activin E as an anti-metabolic disease factor. However, some studies have reported increased hepatic expression of Activin E in patients with MASLD or animals, raising the possibility that it may have adverse effects on lipid metabolism [[12], [13], [14]].

Moreover, Activin E has been shown to possess anti-lipolytic activity, and high-fat diet (HFD)-fed Activin E knockout (KO) mice show reduced systemic fat mass along with hepatic fat accumulation [[15], [16], [17]]. However, detailed analyses of MASH-related pathologies, such as hepatic inflammation and fibrosis, have not yet been conducted.

Therefore, to investigate the involvement of Activin E depletion in MASH, we characterized the phenotype of Activin E-KO mice under an HFD. Notably, Activin E-KO mice did not show obesity, despite high-fat feeding, but showed increased liver weight. A histological examination showed fatty liver and MASH-like lesions, suggesting that Activin E-KO mice that consume an HFD may be appropriate as a MASH model. Therefore, Activin E-KO mice may be useful for studying lean-type MASH, providing insights into the pathogenesis of MASH that develops independently of obesity.

## 2 Materials and methods

### 2.1 Animal studies

Activin E-KO (*Inhbe*^*−/−*^) mice were generated as described previously [9]. The mice were maintained under a 12-h light–dark cycle at 23 °C ± 2 °C and allowed free access to standard laboratory food (CE-2; Clea Japan, Tokyo, Japan) and water. The mice were started on an HFD (No. D12492; Research Diets, New Brunswick, NJ, USA) at 13–15 weeks of age. The body weight of the mice was measured weekly at a fixed time point following a 2-h fasting period. Blood samples were collected from the axillary arteriovenous veins of mice anesthetized with 2 % sevoflurane (Zoetis, Tokyo, Japan), followed by tissue collection after euthanasia via cervical dislocation. Experimental procedures and the care of the mice were in accordance with the requirements of the Institutional Animal Care Committee at Kitasato University or Nagahama Institute of Bio-Science and Technology and in compliance with U.S. National Institutes of Health guidelines (approval number. 18–131, 19-012, 095).

### 2.2 General histological procedures

Tissue fixation using Bouin's fluid, paraffin section preparation, hematoxylin and eosin staining, and immunohistochemical procedures were performed as described previously [9].

### 2.3 Sirius Red staining

To perform Sirius Red staining, deparaffinized sections were incubated with Sirius Red solution for 1 h. After rinsing with acetic acid, the sections were dehydrated, cleared, and mounted for microscopic analysis.

### 2.4 Oil Red O staining

Oil Red O staining was performed as follows. Liver tissue was fixed overnight in 10 % formalin neutral buffer solution, rinsed with phosphate-buffered saline (PBS), and progressively immersed in 10 %–30 % sucrose solution in PBS at 4 °C. The tissue was then embedded in OCT compound (Sakura Finetek) and rapidly frozen in liquid nitrogen. Ten-micrometer-thick sections were cut using a cryostat, adhered to glass slides, and stained with Oil Red O according to previously established protocols.

Alternatively, liver fragments fixed with 10 % formalin neutral buffer solution were rinsed with PBS and stained with Oil Red O. After 2 h, the tissues were washed with 60 % isopropanol and extracted in 100 % isopropanol (20 μL of solution per mg of tissue). The absorbance at 490 nm was measured using a microplate reader.

### 2.5 Immunohistochemistry

To perform immunohistochemistry, a rabbit polyclonal anti-Ucp1 antibody (3 μg/mL, ab10983; Abcam, Cambridge, UK) or anti-F4/80 antibody (1:250, 70076; Cell Signaling Technology, Danvers, MA, USA) was reacted with deparaffinized sections overnight at 4 °C and visualized with 3,3′-diaminobenzidine tetrahydrochloride using a Histofine Simple Stain MAX-PO kit (Nichirei, Tokyo, Japan) as described previously [9].

### 2.6 Adipocyte size analysis

To analyze adipocyte size, we used hematoxylin and eosin-stained WAT. All images of the hematoxylin-and eosin-stained sections were obtained with an all-in-one fluorescence microscope (BZ-X710; Keyence, Osaka, Japan) using a Plan Apochromat 20 × objective (NA0.75, BZ-PA20, Keyence). The cell size was analyzed using ImageJ software (version 1.52, U.S. National Institutes of Health, Bethesda, MD, U.S.A.). The density and distribution of adipocytes were subsequently calculated.

### 2.7 Hepatic lipid area quantification

To analyze the area of lipidation in hepatocytes in the liver, we captured five randomly selected views in each mouse from hematoxylin and eosin-stained liver sections (magnification: 15× 4), ensuring unbiased sampling across the liver tissue. The captured images were binarized and analyzed using ImageJ software to quantify the fatty area. The fatty area (%) was calculated using the following formula: total area of vacuoles created by fat droplets (μm^2^)/total area of liver parenchyma and stroma excluding blood vessels (μm^2^) × 100. Standardized thresholding was applied to the binarized images to identify fat vacuoles consistently across different samples. The total area of the liver parenchyma and stroma was carefully measured, excluding blood vessels, to ensure an accurate calculation of the fatty area. The numerical data from each field of view were analyzed statistically.

### 2.8 Hepatic crown-like structure quantification

To analyze the number of hepatic crown-like structures (hCLSs), liver sections were immunohistochemically stained with an anti-F4/80 antibody, and we photographed 10 fields of view selected without preference (magnification: 15× 10) for each mouse to ensure unbiased sampling. Binarized images were analyzed using ImageJ software to identify and quantify hCLSs. The number of hCLSs (/mm^2^) was calculated using the following formula: number of hCLSs in one field of view/total area of liver parenchyma and stroma excluding blood vessels (μm^2^) × 10^6^. Automatic thresholding and standardization across all images were implemented to ensure consistent identification of hCLSs. Blood vessels were excluded from the analysis using the region of interest tools in ImageJ software to ensure that only the liver parenchyma and stroma were considered. The numerical data obtained from each field of view were analyzed statistically.

### 2.9 Adipose tissue crown-like structure quantification

To analyze the number of crown-like structures (CLSs) in adipose tissue, all images of the sections stained immunohistochemically using anti-F4/80 antibody were obtained with an all-in-one fluorescence microscope (BZ-X710; Keyence) using a Plan Apochromat 10 × objective (NA0.45, BZ-PA10; Keyence). Five fields of view selected without preference were captured for each WAT (inguinal subcutaneous: i, epididymis: e, perirenal: p, mesenteric: m) and brown adipose tissue (BAT) section. The captured images were binarized and analyzed using ImageJ software to quantify the CLSs. The number of CLSs (/mm^2^) was calculated using the following formula: number of CLSs found in one field of view/total area of WAT or BAT (μm^2^) × 10^6^. The fields of view were selected without preference to reduce sampling bias and ensure representative analysis across tissue samples. Standardized thresholding was applied across all of the images to identify CLS regions consistently. The total area of the WAT or BAT was calculated carefully, excluding any irrelevant tissue types or artifacts to ensure accurate measurement. The numerical data from each field of view were subjected to statistical analysis.

### 2.10 Fibrotic area in the liver

To analyze the fibrotic area in liver tissue, 10 fields of view (magnification: 15× 20) selected without preference from Sirius Red-stained liver sections from each mouse were photographed, focusing on the central vein and portal vein regions to ensure a comprehensive analysis. Binarized images were analyzed using ImageJ software to quantify fibrosis. The fibrotic area (%) was calculated using the following formula: total area of fibrosis (μm^2^) per field of view/total area of liver parenchyma and stroma excluding fibers around blood vessels and blood vessels themselves (μm^2^) × 100. Automatic and standardized thresholding was applied across all images to ensure consistent detection of fibrotic regions. Fibers surrounding blood vessels were manually or automatically excluded from the analysis using the region of interest tools from ImageJ software to ensure accurate measurement of the liver parenchyma and stroma. The central and portal vein regions were analyzed independently to assess differences in the distribution of fibrosis, with clear documentation of the regions being analyzed. The numerical data obtained from each field of view were analyzed statistically.

### 2.11 Histological evaluation of MASH using the NAFLD activity score

The assessment of MASH pathology was performed according to the NAFLD activity score (NAS) proposed by Kleiner et al. [18]. Steatosis (NAS: 0–3), inflammation (NAS: 0–3), and hepatocellular ballooning (NAS: 0–2) were evaluated, and the total NAS was calculated. A total score of ≤2 was considered simple steatosis, a score of 3–4 was considered borderline, and a score of ≥5 was considered diagnostic of MASH.

### 2.12 Blood examination

Blood samples were incubated at 25 °C for 2 h, centrifuged at 3000 rpm for 10 min, and stored at −80 °C until use. Serum biochemical indices were determined using various measuring regents (Fujifilm Wako Pure Chemical, Osaka, Japan) in a 7180 Clinical Analyzer (Hitachi High-Tech, Tokyo, Japan). The reagents used to measure alanine transaminase (ALT), aspartate transaminase (AST), lactate dehydrogenase (LDH), nonesterified fatty acids (NEFAs), and triglycerides (TGs) were L-Type ALT J2, L-Type AST J2, L-type LD J, NEFA-HR, and L-Type TG M, respectively.

Serum insulin concentrations were measured using an ultrasensitive mouse insulin ELISA kit (cat No. M1104; Morinaga Institute of Biological Science, Yokohama, Japan). In the insulin tolerance test, the mice were anesthetized, and 2 mU insulin (Novolin 30R; Novo Nordisk, Denmark) per 1 g body weight was administered intraperitoneally after 4 h of fasting. Blood was drawn from the caudal vein at the indicated times. Blood glucose concentrations were measured using a Medisafe test strip and Blood Glucose Monitoring System (Terumo, Tokyo, Japan).

Adipose tissue insulin resistance was evaluated using the adipose insulin resistance index (Adipo-IR), which was calculated as the product of fasting insulin and fasting free fatty acid concentrations, as previously described [19].

### 2.13 RNA sequencing

Isogen II (Nippon Gene, Tokyo, Japan) was used to extract total RNA from the liver of Activin E-KO mice that consumed an HFD for 6 weeks. The transcriptomes were sequenced on the Illumina NovaSeq X Plus platform (Illumina, San Diego, CA, USA), and the resulting data were analyzed by Rhelixa (Tokyo, Japan). Adapter sequences and low-quality bases from FASTQ files were trimmed using Trimmomatic software (version 0.38). The trimmed reads were subsequently aligned to the reference genome using HISAT2 (version 2.1.0). In each sample, transcript/gene read counts, fragments per kilobase of transcript per million mapped reads, and transcripts per kilobase million were calculated.

Differential expression was analyzed using DESeq2 (version 1.24.0) and compared with that of the controls, as specified. Differentially expressed genes were identified using the thresholds of |log2-fold change| > 1 and an adjusted *p* < 0.05, as determined by the Benjamini–Hochberg method for false discovery rate correction. Gene ontology (GO) enrichment analysis of the differentially expressed genes was conducted using GOATOOLS software (version 1.1.6), with *p* values adjusted for multiple testing using the Benjamini–Hochberg method. The RNA sequence data from this study have been deposited in ArrayExpress under the accession number E-MTAB-14962.

### 2.14 Statistical analysis

The results are expressed as the mean ± standard error of the mean (SEM). Gene expression data were log-transformed to provide an approximation of a normal distribution before analyses. Datasets were compared using the Student *t*-test. The Dunnett multiple comparisons test was used to compare data between groups. Statistical analyses were conducted using Prism 10 software (GraphPad Software, San Diego, CA, USA). Differences of *p* < 0.05 were considered significant.

## 3 Results

### 3.1 Liver weight is increased in Activin E-KO mice that consumed an HFD

**Fig. 1:**
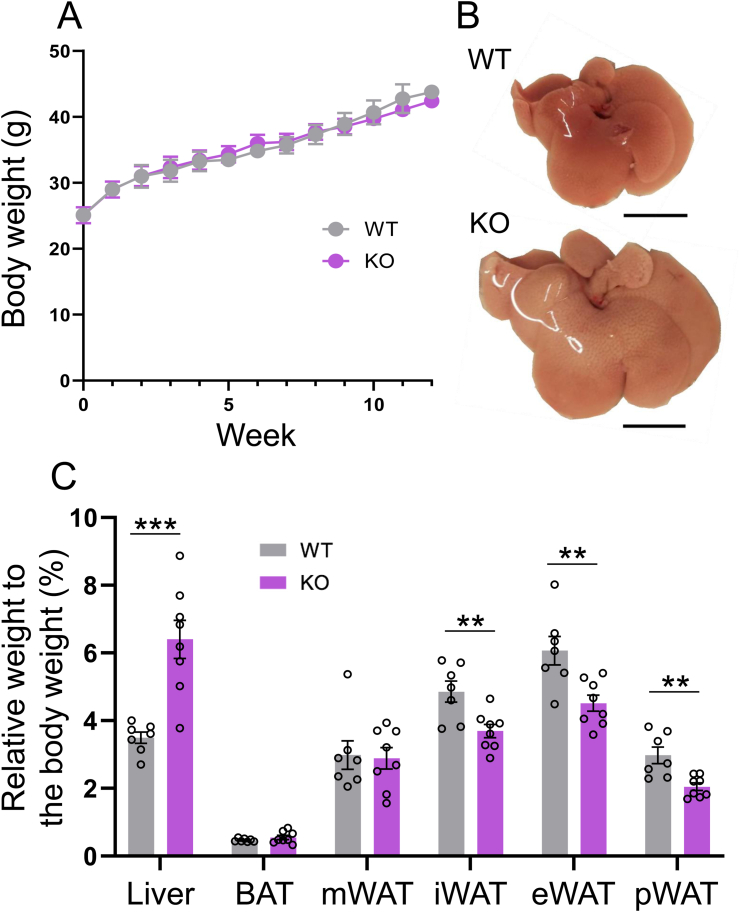
**Body and tissue weights of Activin E-KO mice that consumed an HFD.** Activin E-knockout (KO) mice fed a high-fat diet (HFD) showed no significant difference in body weight compared with WT mice, but they showed hepatomegaly and reduced adipose tissue mass. (A) Growth curve of male Activin E-KO mice that consumed an HFD. The mice were started on an HFD at 13–15 weeks of age and weighed weekly for 12 weeks. Wild type (WT), n = 7; KO, n = 8. (B) Gross appearance of the liver from mice that consumed an HFD for 8 weeks. Representative data are shown. Bar, 1 cm. (C) Liver and adipose tissue weights from male Activin E-KO mice that consumed an HFD for 10–12 weeks. Relative tissue weight is expressed as the percentage of total body weight. m, mesenteric; i, inguinal; e, epididymal; p, perirenal. Results are shown as the mean ± SEM. WT, n = 7; KO, n = 8. ∗∗*p* < 0.01, ∗∗∗*p* < 0.001.

After consuming an HFD for 12 weeks, there was no significant difference in body weight or food intake between male Activin E-KO mice and male wild type (WT) mice (Fig. 1A, Supplementary Fig. 1). However, a macroscopic examination showed that the livers of Activin E-KO mice were significantly larger than those of WT mice (Fig. 1B), with a concomitant increase in liver weight (Fig. 1C). In contrast, the weights of epididymal, inguinal, perirenal, and adipose tissues were lower in Activin E-KO mice than in WT mice (Fig. 1C). Even after consuming an HFD for 6 weeks, the liver weight of Activin E-KO mice was greater than that of WT mice. The weight of each type of adipose tissue in Activin E-KO mice tended to be less than that in WT mice (Supplementary Fig. 2).

### 3.2 MASH-like phenotype in the liver of Activin E-KO mice that consumed an HFD

**Fig. 2:**
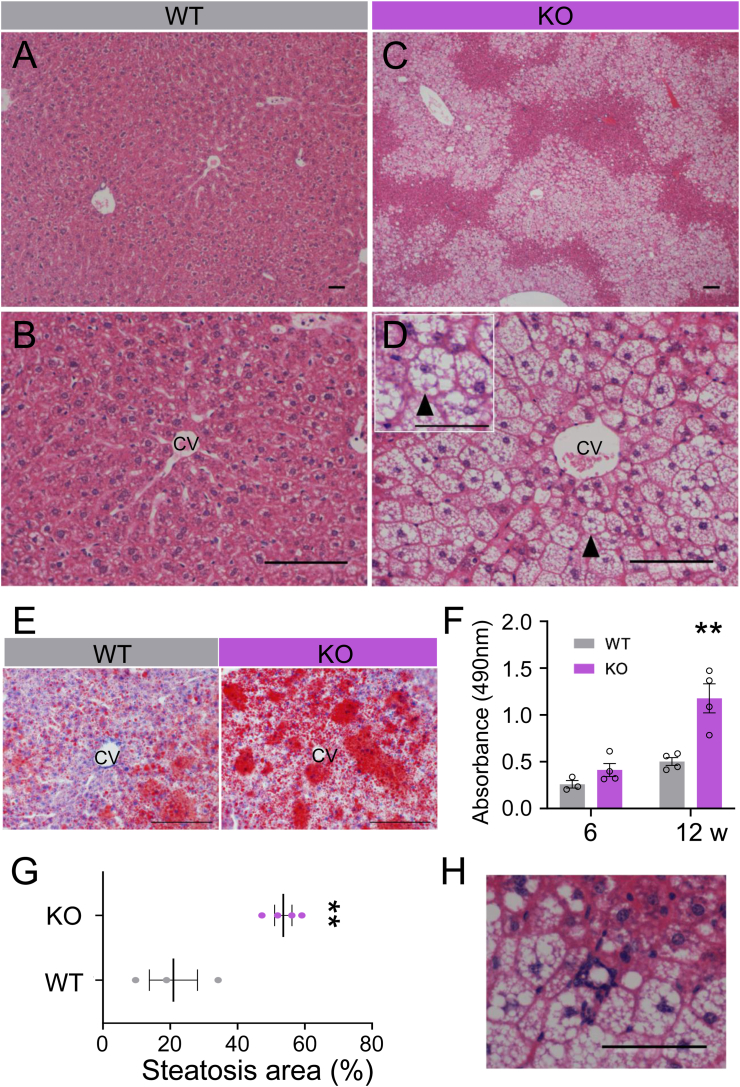
**Characteristics of the liver of Activin E-KO mice.** Liver histology showed that HFD-fed Activin E-KO mice developed hepatic steatosis. (A–D) Hematoxylin and eosin staining of liver sections from male Activin E-KO mice that consumed an HFD for 8 weeks. Representative data are shown. Arrowheads, Mallory–Denk bodies; bars, 100 μm. (E) Oil Red O staining of frozen liver sections from male Activin E-KO mice that consumed an HFD for 6 or 12 weeks. Representative data are shown. Bars, 20 μm. (F) Quantification of Oil Red O staining from male Activin E-KO mice that consumed an HFD for 6 or 12 weeks. Oil Red O was extracted from the stained livers with isopropanol, and the absorbance of the dye solution was measured. WT, n = 3; KO, n = 4. ∗∗*p* < 0.01. (G) Quantification of the steatotic area in the liver of mice that consumed an HFD for 8 weeks. Results are shown as the mean ± SEM. WT, n = 3; KO, n = 4. ∗∗*p* < 0.01. (H) Hematoxylin and eosin staining of hepatic crown-like structures in the liver of male Activin E-KO mice that consumed an HFD for 8 weeks. Representative data are shown. Bar, 50 μm. CV, central vein; HFD, high-fat diet; KO, knockout; w, weeks; WT, wild type.

**Fig. 3:**
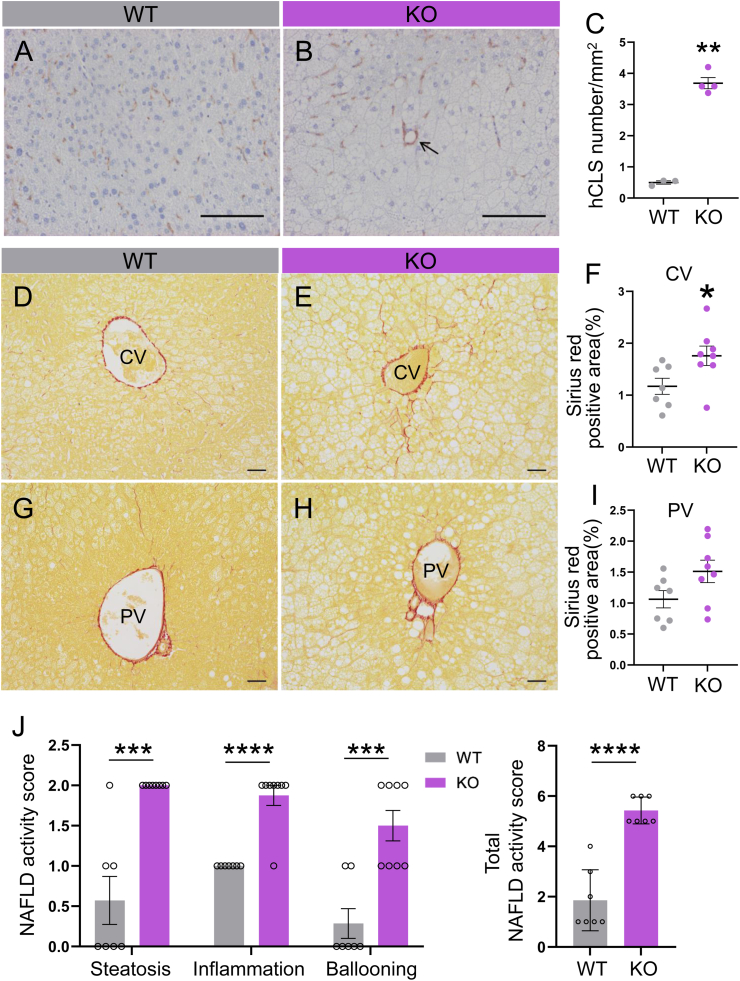
**Infiltration of macrophages and fibrosis in the liver of Activin E-KO mice.** Livers of HFD-fed Activin E-KO mice showed an increased amount of hCLSs and fibrosis. Scoring using the NAS demonstrated that these mice developed MASH. (A, B) F4/80 staining of the liver sections from male Activin E-KO mice that consumed an HFD for 8 weeks. Representative data are shown. Bars, 100 μm. (C) Quantification of the number of hCLSs in the liver of mice. WT, n = 3, KO, n = 4. ∗∗*p* < 0.01. (D, E, G, H) Sirius Red staining of liver sections from male Activin E-KO mice that consumed an HFD for 10–12 weeks. Representative data are shown. Bars, 50 μm. (F, I) Quantification of peri-CV or peri-PV Sirius Red positive areas in the liver of mice. WT, n = 7; KO, n = 8. ∗*p* < 0.05. (J) Metabolic dysfunction-associated steatotic liver disease/non-alcoholic fatty liver disease activity score of the liver of mice. Left panel: individual scores for steatosis, inflammation, and ballooning; right panel: total NAS calculated for each mouse. Results are shown as the mean ± SEM. WT, n = 7; KO, n = 8. ∗∗*p* < 0.01, ∗∗∗*p* < 0.001. CV, central vein; hCLSs, hepatic crown-like structures; HFD, high-fat diet; KO, knockout; PV, portal vein; WT, wild type.

A histological examination showed the presence of numerous ballooned hepatocytes with lipid droplets in the central vein region of the liver in Activin E-KO mice that consumed an HFD, in contrast to the hepatocytes observed in WT mice (Fig. 2A–D). Oil Red O staining of frozen liver sections confirmed fatty degeneration (Fig. 2E and F), and the area occupied by lipid droplets was significantly greater in Activin E-KO mice than in WT mice. Additionally, Mallory–Denk-like bodies were observed in ballooned cells in Activin E-KO mice (Fig. 2D). hCLSs, which were characterized by macrophages encircling lipid-laden hepatocytes, were also observed (Fig. 2H). Immunostaining with an anti-F4/80 antibody showed a significantly higher number of hCLSs in Activin E-KO mice than in WT mice (Fig. 3A–C). Fibrosis surrounding the central vein was more extensive in Activin E-KO mice after consuming an HFD for 10–12 weeks than in WT mice (Fig. 3D–F), and there was a nonsignificant tendency toward increased fibrosis around the portal vein (Fig. 3G–I). The lesions observed in Activin E-KO mice that consumed an HFD are consistent with those observed in previous studies.

Furthermore, evaluation using the NAS score showed MASH-like lesions in these mice. When the total NAS was calculated for each mouse, all Activin E-KO mice scored ≥5, which fulfilled the diagnostic criteria for MASH (Fig. 3J).

### 3.3 MASH-related gene expression in the liver of Activin E-KO mice that consumed an HFD

**Fig. 4:**
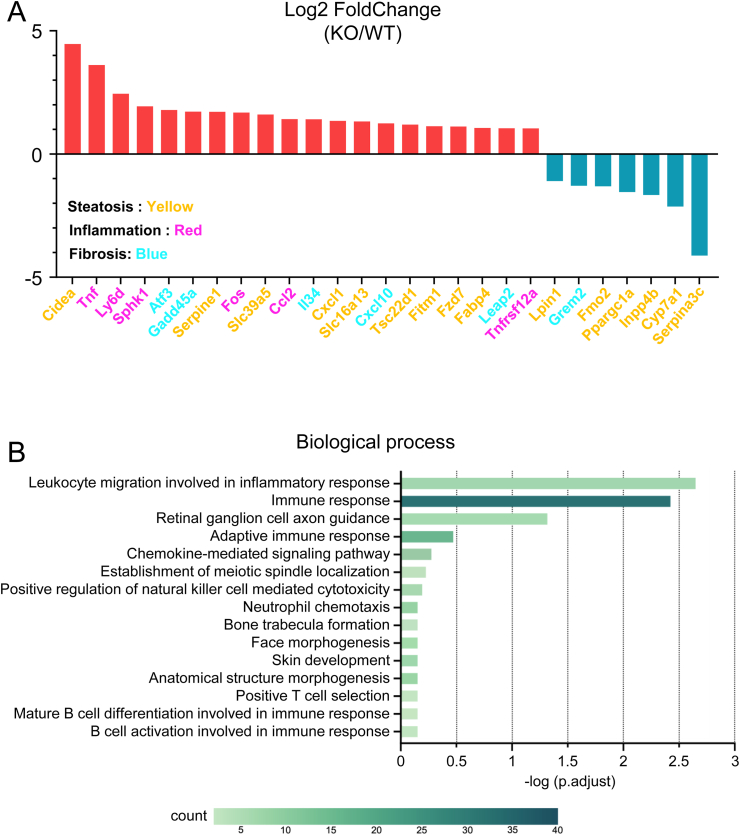
**RNA sequencing analysis of the liver of Activin E-KO mice that consumed an HFD.** RNA sequencing analysis of the liver from HFD-fed Activin E-KO mice showed altered expression of genes associated with MASH. The livers of KO mice that consumed an HFD for 6 weeks were subjected to RNA sequencing analysis. WT, n = 3; KO, n = 3. (A) Relative expression levels of genes associated with inflammation, fibrosis, fatty liver, lipid metabolism, and metabolic dysfunction-associated steatohepatitis. (B) Bar graph created using the top 15 gene ontology terms for biological processes obtained from the gene ontology analysis, starting with the smallest *p* value. HFD, high-fat diet; KO, knockout; WT, wild type.

An RNA sequencing (RNA-seq) analysis of total RNA from the liver of Activin E-KO mice that consumed an HFD for 6 weeks showed differential expression of various genes associated with inflammation, fibrosis, and steatosis (Fig. 4A). The GO analysis showed significant upregulation of terms related to leukocyte migration in the inflammatory response (GO:0002523) and immune response (GO:0006955) (Fig. 4B). Genes associated with MASH features, including lipid accumulation (e.g., *Cidea*, *Serpine1*, *Slc39a5*, *Slc16a13*, *Inpp4b*, *Lipin1*, *Cxcl1*, *Tsc22d1*, *Fitm1*, *Fzd7*, *Fabp4*, *Fmo2*, *Ppargc1a*, *Cyp7a1*, and *Serpina3c*), inflammation (e.g., *Fos*, *Tnfrsf12a*, *Ly6d*, *Ccl2*, *Sphk1*, and *Tnf*), fibrosis (e.g., *Atf3*, *Gadd45a*, *Cxcl10*, *Leap2*, *Grem2*, and *Il34*), insulin resistance, and lipid metabolism, were elevated. Additionally, genes including *Gadd45a* (for a DNA damage response protein) and *Fzd7* (a G protein-coupled receptor encoding gene), of which the human versions of both are elevated in patients with MASH [20,21], were significantly upregulated in Activin E-KO mice. These findings suggest that Activin E-KO mice show MASH-like symptoms and altered lipid metabolism and insulin resistance. In contrast, the expression of hepatic *de novo* lipogenesis-related genes, including lipogenic transcription factors (*Srebf1* and *Mlxipl*), fatty acid-synthesizing enzymes (*Acly*, *Acaca*, *Fasn*, *Scd1*, and *Elovl6*), and a triglyceride-synthesizing enzyme (*Dgat2*), was not significantly different between Activin E-KO and wild-type mice (Supplementary Table 1).

### 3.4 Fat accumulation is reduced in WAT in Activin E-KO mice

**Fig. 5:**
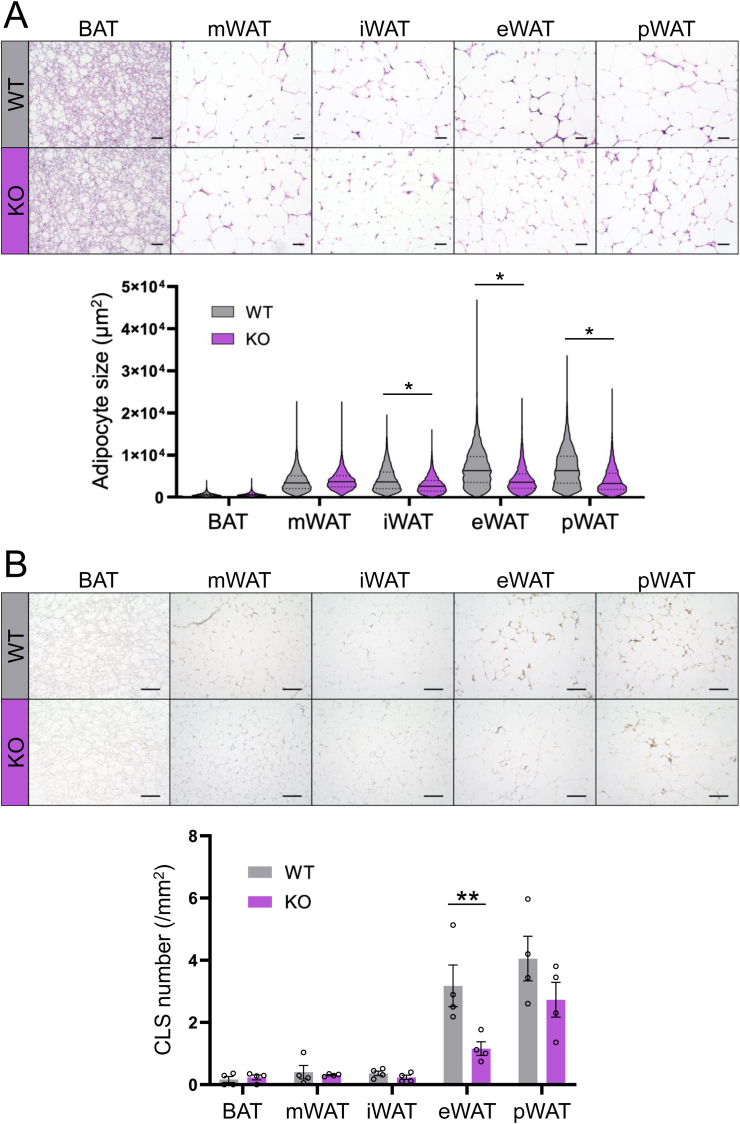
**Histological analysis of adipose tissue from Activin E-KO mice fed an HFD.** Adipose tissue in HFD-fed Activin E-KO mice showed smaller adipocytes and fewer CLSs than WT mice. (A) Upper panel: hematoxylin and eosin staining of adipose tissue sections from male Activin E-KO mice after consuming an HFD for 10–12 weeks. Representative data are shown. Bars, 100 μm. Lower panel: quantification of the cell size in adipose tissue from the mice. (B) Upper panel: F4/80 immunostaining of adipose tissue sections from male Activin E-KO mice after consuming an HFD for 10–12 weeks. Representative data are shown. Bars, 200 μm. Lower panel: quantification of the number of CLSs in adipose tissue from the mice. The cell size and number of CLSs were quantified using ImageJ software. n = 4 in each group. Results are shown as the mean ± SEM. Adipose cell size was compared using the Mann–Whitney *U* test. ∗*p* < 0.05, ∗∗*p* < 0.01. CLSs, crown-like structures; HFD, high-fat diet; i, inguinal; KO, knockout; m, mesenteric; p, perirenal; WT, wild type.

A histological analysis of WAT showed a lower WAT weight in Activin E-KO mice than in WT mice (Fig. 1C). A violin plot shows that adipocytes in adipose tissues were smaller in Activin E-KO mice than in WT mice, except in BAT (Fig. 5A). Immunohistochemistry with an anti-F4/80 antibody showed a general trend toward less CLSs in WAT of Activin E-KO mice than in that of WT mice, with significantly less CLSs specifically in epidydimal WAT (Fig. 5B). These findings suggest that lipid accumulation is suppressed in WAT of Activin E-KO mice, in contrast to WT mice, and that an HFD promotes hypertrophy of adipocytes and subsequent inflammation.

### 3.5 MASH-like findings of blood parameters and insulin resistance

**Fig. 6:**
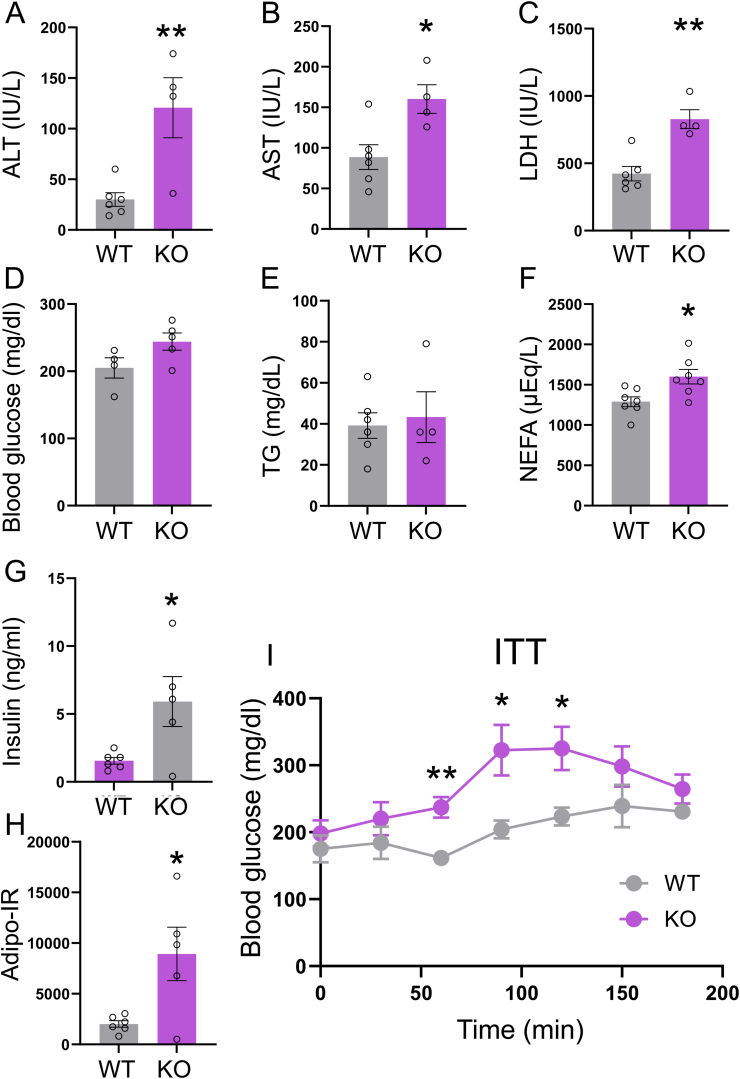
**Blood parameters of Activin E-KO mice that consumed an HFD.** Serum biochemical analyses showed that HFD-fed Activin E-KO mice developed liver injury and insulin resistance. Serum from Activin E-KO male mice that consumed an HFD for 10–12 weeks was subjected to blood chemistry. (A) Alanine transaminase (ALT), (B) aspartate transaminase (AST), (C) lactate dehydrogenase (LDH), (D) blood glucose, (E) triglycerides (TGs), (F) nonesterified fatty acids (NEFAs), (G) insulin, and (H) the adipose insulin resistance (Adipo-IR) index. n = 4–7 in each group. ∗*p* < 0.05, ∗∗*p* < 0.01. (I) Insulin tolerance tests in male Activin E-KO mice that consumed an HFD for 6 weeks. WT, n = 4; KO, n = 5. Values are the mean ± SEM. *∗p* < 0.05, ∗∗*p* < 0.01. HFD, high-fat diet; KO, knockout; WT, wild type.

Activin E is known to enhance insulin sensitivity. Therefore, we hypothesized that deficiency of Activin contributes to the development of insulin resistance in adipose tissue. We conducted biochemical analyses to assess MASH-like symptoms and insulin sensitivity in Activin E-KO mice fed an HFD. Serum concentrations of AST, ALT, and LDH, which are markers of hepatic injury, were significantly higher in Activin E-KO mice than in WT mice (Fig. 6A–C). No significant differences in blood glucose or TG concentrations were observed between the groups (Fig. 6D and E). However, serum concentrations of NEFAs and insulin and the Adipo-IR index were significantly higher in Activin E-KO mice than in WT mice (Fig. 6F–H). Activin E-KO mice fed an HFD showed reduced insulin sensitivity compared with WT mice fed an HFD (Fig. 6I). These findings suggest that Activin E deficiency leads to elevated serum concentrations of free fatty acids and insulin resistance, reflecting suppressed lipid metabolism/accumulation in WAT of Activin E-KO mice fed an HFD.

Generally, excessive fat accumulation in adipocytes leads to insulin resistance. However, in Activin E-KO mice fed an HFD, we speculate that they show early-stage insulin resistance during high-calorie loading because of Activin E signal deficiency. Therefore, the deletion of Activin E inhibits the physiological fat accumulation process.

### 3.6 Sex-specific differences in response to an HFD in Activin E-KO mice

**Fig. 7:**
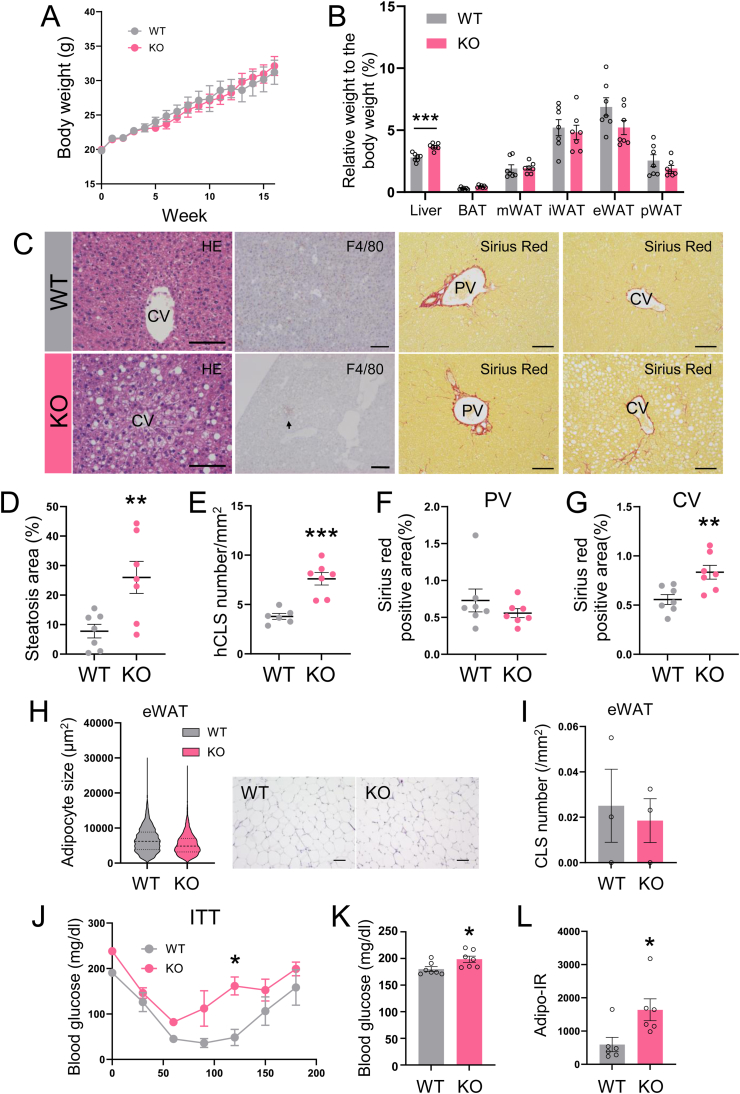
**Phenotypic analysis of hepatic pathology and adipose tissue in female HFD-fed Activin E-KO mice.** Female Activin E-KO mice required a longer duration of HFD feeding to develop MASLD than male mice. (A) Growth curve of female Activin E-KO mice that consumed an HFD. The mice were started on an HFD at 13–15 weeks of age and weighed weekly for 16 weeks. WT, n = 10; KO, n = 10. (B) Liver and adipose tissue weights of female Activin E-KO mice that consumed an HFD for 16 weeks. Relative tissue weight is expressed as the percentage of total body weight. WT, n = 7; KO, n = 7. (C) Hematoxylin and eosin staining, F4/80 staining, and Sirius Red staining of liver sections from female Activin E-KO mice that consumed an HFD for 16 weeks. Representative data are shown. bars, 100 μm. (D) Quantification of the steatotic area in the liver of female Activin E-KO mice. WT, n = 7; KO, n = 7. (E) Quantification of the number of hCLS in the liver of female Activin E-KO mice. WT, n = 6; KO, n = 7. (F, G) Quantification of peri-CV or peri-PV Sirius Red-positive areas in the liver of female Activin E-KO mice. WT, n = 7; KO, n = 7. (H) Right panel: hematoxylin and eosin staining of epididymal WAT sections from female Activin E-KO mice after consuming an HFD for 16 weeks. Representative data are shown. Bars, 100 μm. Left panel: quantification of cell size in epididymal WAT from female Activin E-KO mice. n = 3 in each group. Adipose cell size was compared using the Mann–Whitney *U* test. (I) Quantification of the number of CLSs in epididymal WAT from female Activin E-KO mice after consuming an HFD for 16 weeks. The cell size and CLS number were quantified using ImageJ software. n = 3 in each group. (J) Insulin tolerance tests in female Activin E-KO mice that consumed an HFD for 10 weeks. (K, L) Serum from female Activin E-KO mice that consumed an HFD for 16 weeks was subjected to blood chemistry. (D) Blood glucose concentrations. (J) Adipo-IR index. Results are shown as the mean ± SEM. n = 6–7 in each group. ∗*p* < 0.05, ∗∗*p* < 0.01, ∗∗∗*p* < 0.001. Adipo-IR, adipose insulin resistance; CLSs, crown-like structures; CV, central vein; e, epididymal; hCLSs, hepatic crown-like structures; HFD, high-fat diet; i, inguinal; KO, knockout; m, mesenteric; p, perirenal; PV, portal vein; WT, wild type.

To evaluate the effect of Activin E deficiency in female mice, we analyzed the phenotype of HFD-fed female Activin E-KO mice. A sex-specific difference was observed in the hepatic phenotypes of Activin E-KO mice fed an HFD, with female mice showing milder hepatic lesions than male mice. After consuming an HFD for 16 weeks, there was no significant difference in body weight between female Activin E-KO mice and female WT mice (Fig. 7A). Unlike HFD-fed male Activin E-KO mice, female Activin E-KO mice did not show an increase in liver weight after 8 weeks of HFD feeding. However, after 12 weeks, a gradual increase in liver weight was observed in female Activin E-KO mice (Supplementary Fig. 3). Following 16 weeks of HFD feeding, liver weight was further elevated, while the weights of various adipose tissues remained unchanged in female Activin E-KO mice (Fig. 7B). A histological analysis of the liver after 16 weeks of HFD showed a significantly greater area occupied by lipid droplets in female Activin E-KO mice than in female WT mice (Supplementary Fig. 4). Although ballooned cells were present in female Activin E-KO mice, they were observed in only a limited number of hepatocytes (Fig. 7C and D). Furthermore, the number of hCLSs was significantly higher in female Activin E-KO mice than in female WT mice (Fig. 7E). While no pericellular fibrosis was detected in the periportal regions (Fig. 7F), fibrotic areas around the central veins were larger in female Activin E-KO mice than in female WT mice (Fig. 7G). These findings suggest that female Activin E-KO mice require a longer duration of HFD feeding to develop MASLD than male Activin E-KO mice.

In contrast to male Activin E-KO mice, female Activin E-KO mice fed an HFD for 16 weeks showed no change in adipocyte size within epididymal WAT (Fig. 7H). The number of CLSs in epididymal WAT also remained unchanged in female Activin E-KO mice compared with female WT mice (Fig. 7I). However, by 10 weeks of HFD feeding, female Activin E-KO mice showed a decrease in insulin sensitivity, similar to that observed in male Activin E-KO mice, compared with female WT mice (Fig. 7J). After 16 weeks of HFD, a biochemical analysis of blood showed that female Activin E-KO mice had significantly higher plasma glucose concentrations (Fig. 7K) and a higher Adipo-IR index than female WT mice (Fig. 7L). This analysis suggested a reduction in insulin sensitivity within adipose tissue.

## 4 Discussion

In this study, we evaluated the utility of Activin E-KO mice fed an HFD as a model of MASH. After 16 weeks of HFD feeding, Activin E-KO mice did not develop obesity but showed reduced WAT mass, along with increased liver weight and hepatic triglyceride content. These findings are consistent with those reported by Adam et al. [15] and Griffin et al. [16]. Such phenotypes are likely due to enhanced lipolysis in adipose tissue caused by the loss of Activin E function, resulting in increased influx of free fatty acids into the liver. Recently, Park et al. [17] also reported a hepatic steatosis model induced by Activin E deficiency, further supporting the role of Activin E in regulating lipid mobilization.

In this study, histological evaluation of liver tissue based on the MASLD activity score showed that the liver of Activin E-KO mice fed an HFD for 16 weeks exhibited histological features consistent with MASH. Additionally, RNA-seq analysis of the liver showed a gene expression profile characteristic of MASH in HFD-fed Activin E-KO mice. These findings demonstrate the utility of HFD-fed Activin E-KO mice as a model for studying MASH.

In humans, the Adipo-IR index, which is calculated by multiplying the fasting free fatty acid concentration by the insulin concentration, indicates insulin resistance in adipose tissue. As glucose tolerance decreases, as observed in diabetes, Adipo-IR values worsen, reflecting increased insulin resistance in adipose tissue [22]. An elevated Adipo-IR has been reported in patients with MASLD/MASH, with higher values associated with worsened AST and ALT concentrations and increased liver fibrosis [23]. The progression of MASLD/MASH is thought to involve not only systemic insulin resistance and glucose intolerance but also deteriorating insulin resistance within adipose tissue.

In the present study, Activin E-KO mice showed hyperinsulinemia and elevated blood NEFA concentrations. These findings indicate that Activin E deficiency impairs lipid metabolism in adipose tissue, thereby reducing insulin action and increasing the influx of free fatty acids into the liver. Hepatic *de novo* lipogenesis contributes to the development of fatty liver [24]. However, our RNA-seq analysis did not show any significant changes in the expression of lipogenesis-related genes in the liver of Activin E-KO mice (Supplementary Table 1). This finding suggests that hepatic steatosis in Activin E-KO mice is unlikely to result from enhanced *de novo* lipogenesis, but is primarily driven by increased fatty acid influx into the liver, which in turn likely contributes to the progression to MASH. Furthermore, the elevated systemic insulin resistance observed in Activin E-KO mice compared with WT mice may be associated with hepatic fibrosis and worsened adipose tissue insulin resistance. Previous studies have shown an increase in beige adipose tissue in Activin E transgenic mice [9,10]. This finding suggests that Activin E may also enhance the expression of Ucp1 in beige adipocytes. This effect is likely mediated through similar signaling pathways similar to those observed in brown adipocytes [11]. These findings suggest that, in addition to the anti-lipolytic effects of Activin E in adipose tissue, it may also promote the browning of WAT and enhance insulin sensitivity.

The degree of CLS formation in adipose tissue has been reported to correlate with the severity and stage of MASLD/MASH (formerly NAFLD/NASH) [25,26]. Interestingly, in our study, despite fewer CLSs observed in WAT in Activin E-KO mice than in WT mice, Activin E-KO mice displayed a more severe MASH phenotype in the liver. This apparent discrepancy may be attributable to impaired lipid storage capacity in adipose tissue due to insulin resistance in Activin E-KO mice. The loss of Activin E's ability to promote adipose tissue browning, namely its effect on increasing Ucp1 expression and enhancing energy expenditure, may further contribute to this insulin-resistant state. Further investigation using recombinant Activin E protein is required to determine its specific roles in adipocytes.

Sex- and age-related differences in the prevalence of MASLD/MASH have been reported in humans, with a higher prevalence observed in middle-aged men and older women [27]. Consistent with this finding, in the present study, female Activin E-KO mice showed a more pronounced MASLD/MASH-like phenotype after 16 weeks of HFD feeding than after 12 weeks of HFD feeding. These findings suggest that extending the duration of HFD feeding or using aged female mice may induce a disease phenotype comparable to that observed in male mice. Estrogen enhances hepatic lipid metabolism via promotion of β-oxidation and suppression of gluconeogenesis, thereby ameliorating insulin resistance [28]. Additionally, estrogen promotes lipolysis [29] and induces browning of WAT through the upregulation of *Ucp1* expression [30]. The sex difference in this study may be explained by the estrogen-mediated upregulation of *Ucp1* expression. Therefore, HFD feeding in aged female mice with diminished estrogen secretion may result in a phenotype similar to that of male Activin E-KO mice.

Taken together, our findings indicate that Activin E is a key metabolic regulator that maintains adipose tissue function, preserves insulin sensitivity, and prevents ectopic lipid accumulation in the liver. The loss of Activin E disrupts lipid storage in WAT, enhances lipolysis, increases hepatic free fatty acid influx, and promotes the development of MASH, even in the absence of obesity. These results highlight a novel mechanism for the pathogenesis of lean-type MASH and emphasize the importance of adipose–liver communication in the progression of liver disease.

Furthermore, our study suggests a sex difference in the function of Activin E. In female KO mice, disease progression was delayed, which may have been attributed to the protective effects of estrogen, such as enhanced lipid metabolism, induction of *Ucp1* expression, and improved insulin sensitivity. These findings indicate that the effect of Activin E deficiency on the susceptibility to MASLD/MASH may be modulated by the hormonal status and aging.

There is a lack of established therapies for MASLD/MASH. Therefore, the Activin E-KO mouse model developed in this study may serve as a useful tool for determining disease mechanisms and identifying novel therapeutic targets, particularly in lean MASH. Future studies using recombinant Activin E protein are required to further clarify the mechanisms of Activin E action in adipocytes.

## 5 Conclusion

This study suggests that Activin E deficiency promotes the development of MASH under HFD feeding. Activin E-KO mice, despite being protected from obesity, show hepatic steatosis and histological features consistent with MASH, including inflammation and fibrosis, thereby presenting with a lean-type MASH phenotype. Furthermore, the observed sex-specific differences resemble the epidemiological features of MASH in humans, providing novel insights that strengthen the value of this model as a representation of human disease. These findings indicate that Activin E is a key regulator of adipose–liver metabolic communication and highlight that Activin E-KO mice are a useful model for determining the pathogenesis of MASH and investigating novel therapeutic strategies.

## CRediT authorship contribution statement

**Maho Sakaki:** Writing – original draft, Visualization, Investigation, Formal analysis, Conceptualization. **Tatsuya Shikata:** Investigation, Formal analysis. **Kensuke Aoki:** Investigation, Formal analysis. **Masaaki Takano:** Investigation, Formal analysis. **Akira Kurisaki:** Validation. **Masayuki Funaba:** Writing – review & editing. **Osamu Hashimoto:** Writing – review & editing, Supervision, Investigation, Conceptualization.

## Funding statement

This work was supported by the 10.13039/501100001691Japan Society for the Promotion of Science 10.13039/501100001691KAKENHI [grant numbers 19K06427, 22K06015, and 25K09444].

## Declaration of competing interest

The authors have nothing to disclose.

## Data Availability

Data will be made available on request.
