# Supplementary material for: Involvement of Activin E depletion in metabolic dysfunction-associated steatohepatitis
Source: Biochem Biophys Rep. 2025 Nov 4;44:102339. doi: 10.1016/j.bbrep.2025.102339 (PMC12634864; doi:10.1016/j.bbrep.2025.102339)
Supplement: Multimedia component 1 [file mmc1.pptx]

## Slide 1
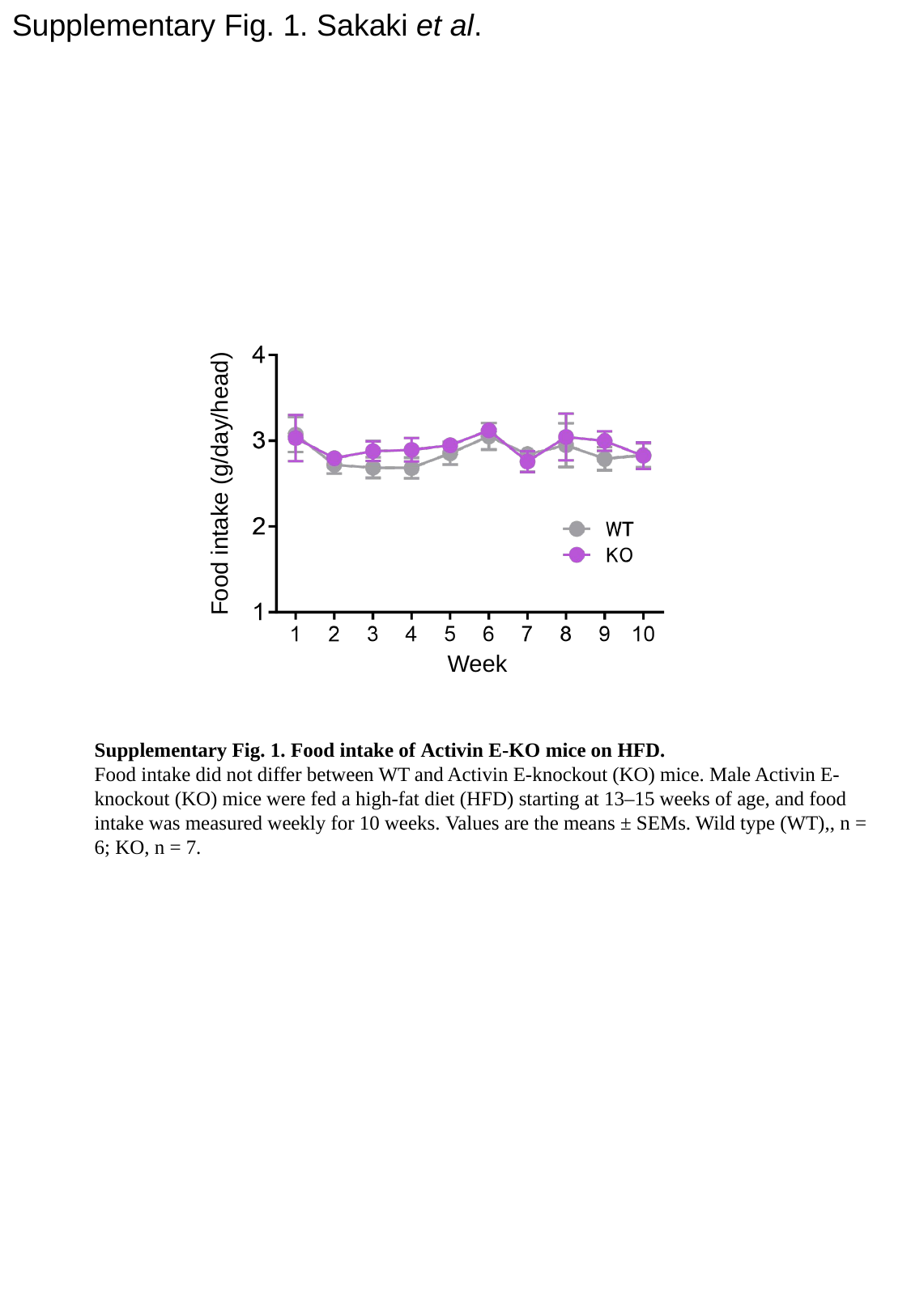

Supplementary Fig. 1. Sakaki et al.
Food intake (g/day/head)
Week
Supplementary Fig. 1. Food intake of Activin E-KO mice on HFD.
Food intake did not differ between WT and Activin E-knockout (KO) mice. Male Activin E-knockout (KO) mice were fed a high-fat diet (HFD) starting at 13–15 weeks of age, and food intake was measured weekly for 10 weeks. Values are the means ± SEMs. Wild type (WT),, n = 6; KO, n = 7.

## Slide 2
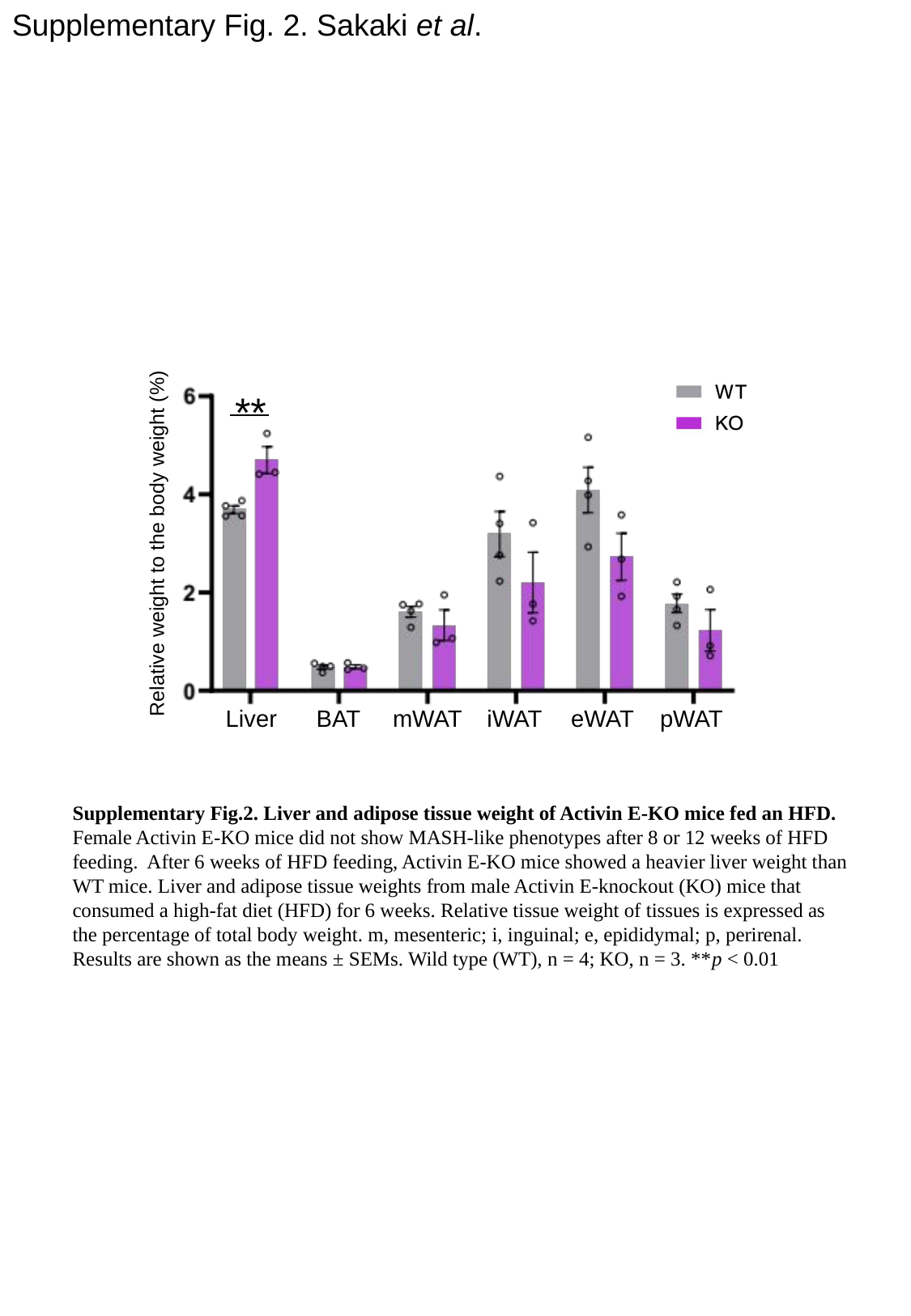

Supplementary Fig. 2. Sakaki et al.
**
Relative weight to the body weight (%)
Liver
BAT
mWAT
iWAT
eWAT
pWAT
Supplementary Fig.2. Liver and adipose tissue weight of Activin E-KO mice fed an HFD.
Female Activin E-KO mice did not show MASH-like phenotypes after 8 or 12 weeks of HFD feeding. After 6 weeks of HFD feeding, Activin E-KO mice showed a heavier liver weight than WT mice. Liver and adipose tissue weights from male Activin E-knockout (KO) mice that consumed a high-fat diet (HFD) for 6 weeks. Relative tissue weight of tissues is expressed as the percentage of total body weight. m, mesenteric; i, inguinal; e, epididymal; p, perirenal. Results are shown as the means ± SEMs. Wild type (WT), n = 4; KO, n = 3. **p < 0.01

## Slide 3
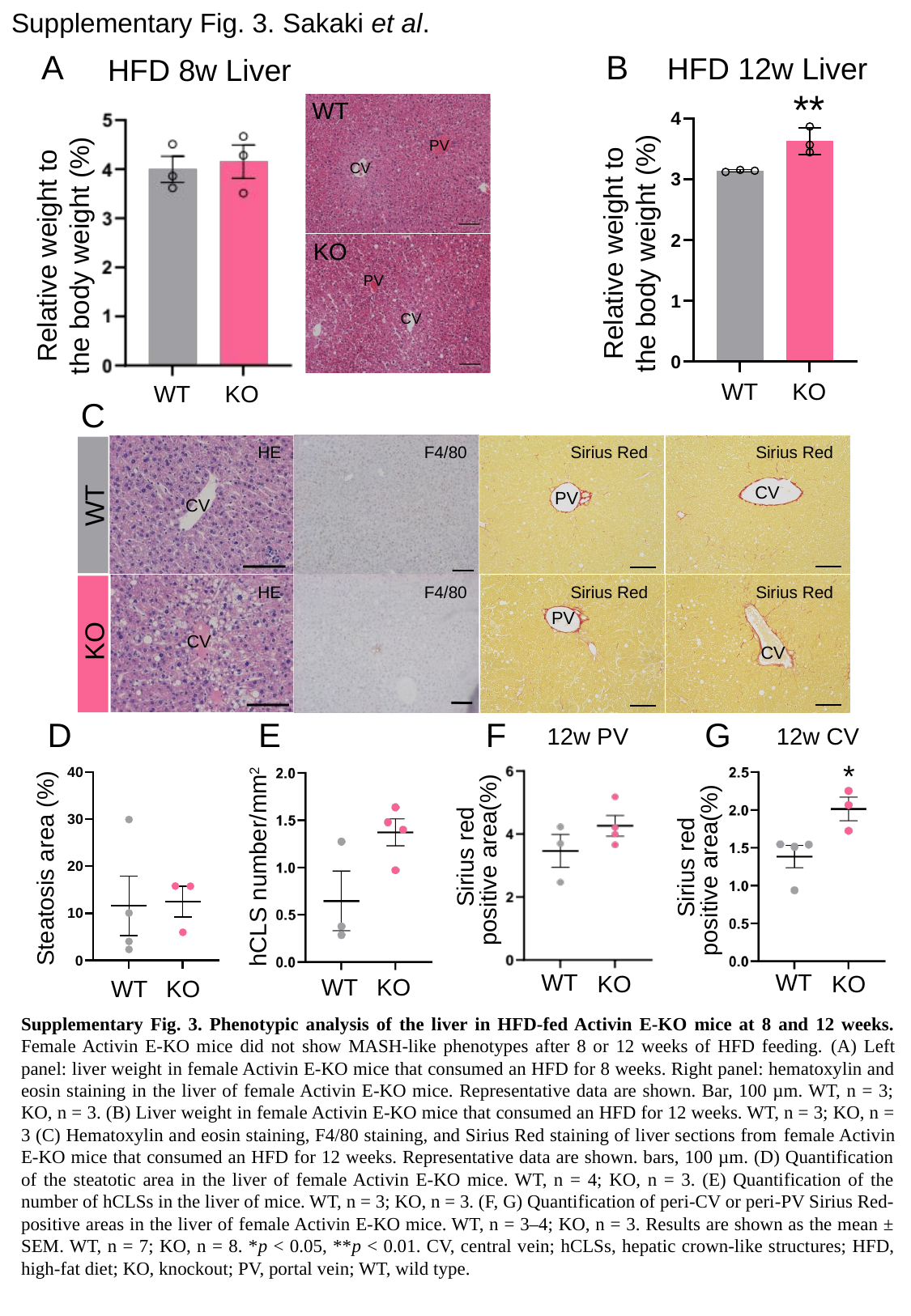

Supplementary Fig. 3. Sakaki et al.
A
B
HFD 12w Liver
HFD 8w Liver
**
WT
PV
CV
Relative weight to the body weight (%)
Relative weight to the body weight (%)
KO
PV
CV
WT
KO
WT
KO
C
HE
F4/80
Sirius Red
Sirius Red
CV
PV
WT
CV
HE
F4/80
Sirius Red
Sirius Red
PV
KO
CV
CV
D
E
F
G
12w PV
12w CV
Sirius red
positive area(%)
Sirius red
positive area(%)
*
hCLS number/mm2
Steatosis area (%)
WT
KO
WT
KO
WT
KO
WT
KO
Supplementary Fig. 3. Phenotypic analysis of the liver in HFD-fed Activin E-KO mice at 8 and 12 weeks. Female Activin E-KO mice did not show MASH-like phenotypes after 8 or 12 weeks of HFD feeding. (A) Left panel: liver weight in female Activin E-KO mice that consumed an HFD for 8 weeks. Right panel: hematoxylin and eosin staining in the liver of female Activin E-KO mice. Representative data are shown. Bar, 100 µm. WT, n = 3; KO, n = 3. (B) Liver weight in female Activin E-KO mice that consumed an HFD for 12 weeks. WT, n = 3; KO, n = 3 (C) Hematoxylin and eosin staining, F4/80 staining, and Sirius Red staining of liver sections from female Activin E-KO mice that consumed an HFD for 12 weeks. Representative data are shown. bars, 100 µm. (D) Quantification of the steatotic area in the liver of female Activin E-KO mice. WT, n = 4; KO, n = 3. (E) Quantification of the number of hCLSs in the liver of mice. WT, n = 3; KO, n = 3. (F, G) Quantification of peri-CV or peri-PV Sirius Red-positive areas in the liver of female Activin E-KO mice. WT, n = 3–4; KO, n = 3. Results are shown as the mean ± SEM. WT, n = 7; KO, n = 8. *p < 0.05, **p < 0.01. CV, central vein; hCLSs, hepatic crown-like structures; HFD, high-fat diet; KO, knockout; PV, portal vein; WT, wild type.

## Slide 4
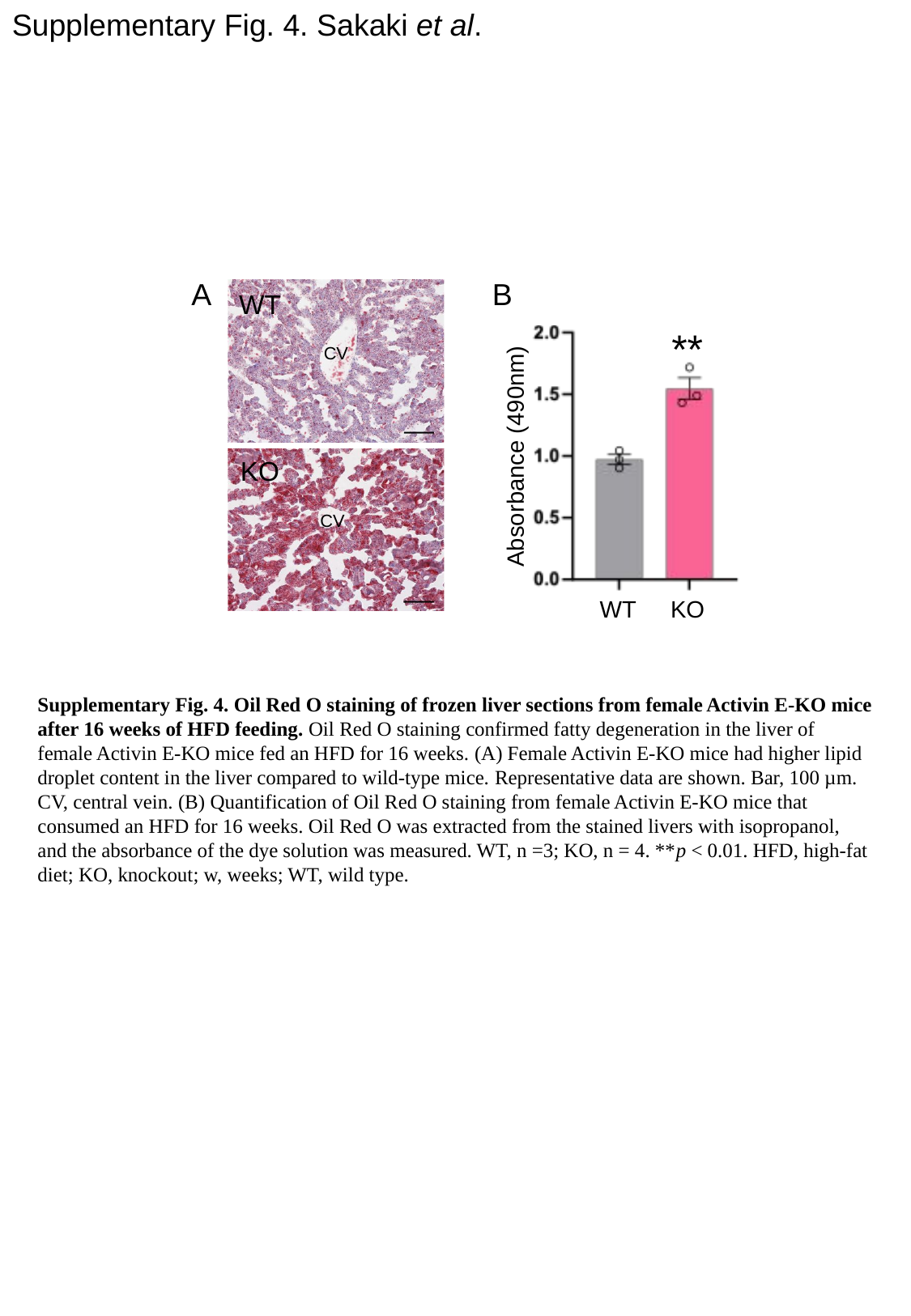

Supplementary Fig. 4. Sakaki et al.
A
B
WT
**
Absorbance (490nm)
WT
KO
CV
KO
CV
Supplementary Fig. 4. Oil Red O staining of frozen liver sections from female Activin E-KO mice after 16 weeks of HFD feeding. Oil Red O staining confirmed fatty degeneration in the liver of female Activin E-KO mice fed an HFD for 16 weeks. (A) Female Activin E-KO mice had higher lipid droplet content in the liver compared to wild-type mice. Representative data are shown. Bar, 100 µm. CV, central vein. (B) Quantification of Oil Red O staining from female Activin E-KO mice that consumed an HFD for 16 weeks. Oil Red O was extracted from the stained livers with isopropanol, and the absorbance of the dye solution was measured. WT, n =3; KO, n = 4. **p < 0.01. HFD, high-fat diet; KO, knockout; w, weeks; WT, wild type.

## Slide 5
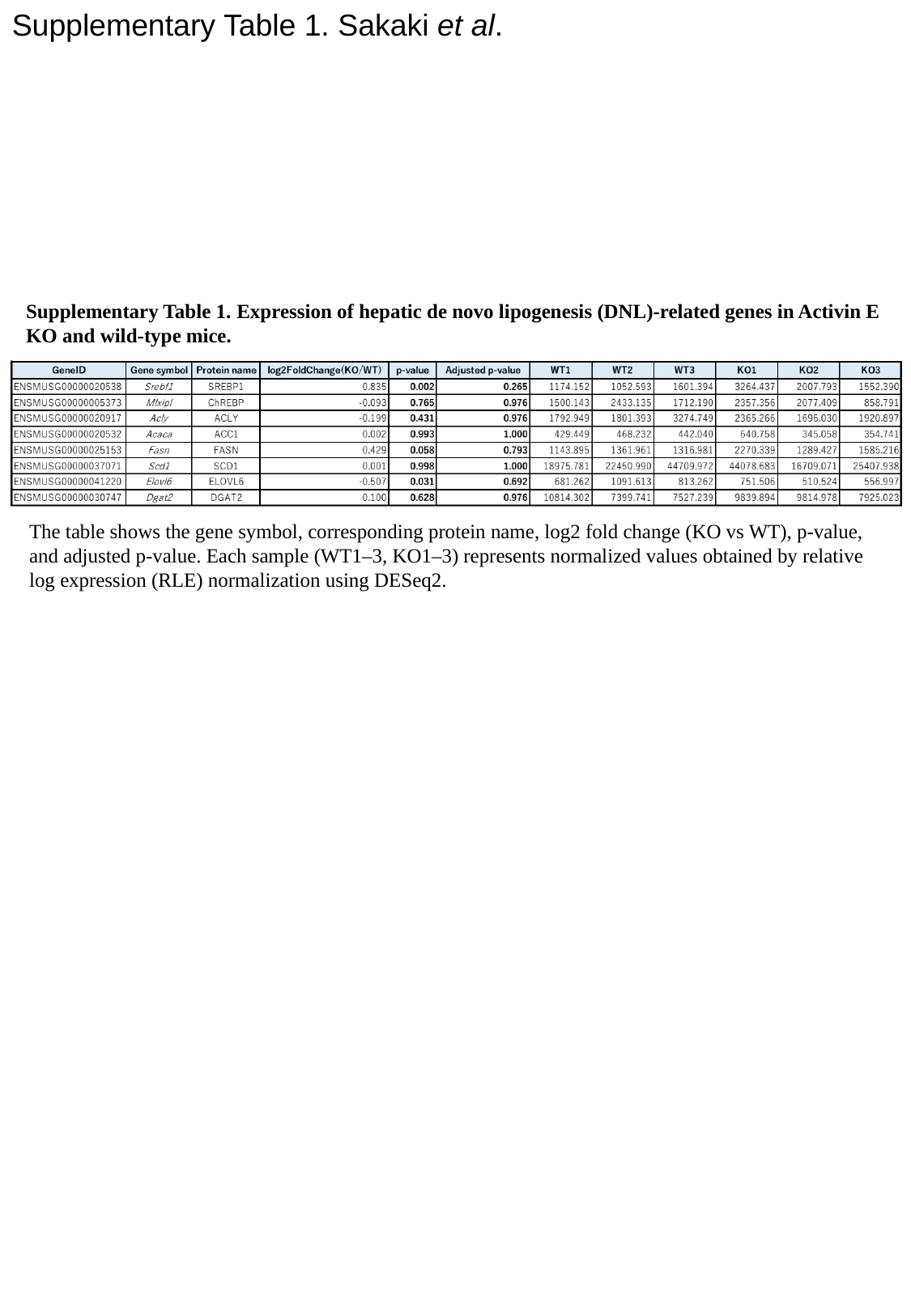

Supplementary Table 1. Sakaki et al.
Supplementary Table 1. Expression of hepatic de novo lipogenesis (DNL)-related genes in Activin E KO and wild-type mice.
The table shows the gene symbol, corresponding protein name, log2 fold change (KO vs WT), p-value, and adjusted p-value. Each sample (WT1–3, KO1–3) represents normalized values obtained by relative log expression (RLE) normalization using DESeq2.
